# Comparative Analysis of Chloroplast Genomes of 19 *Saxifraga* Species, Mostly from the European Alps

**DOI:** 10.3390/ijms26136015

**Published:** 2025-06-23

**Authors:** Zhenning Leng, Zhe Pang, Zaijun He, Qingbo Gao

**Affiliations:** 1 Key Laboratory of Adaptation and Evolution of Plateau Biota, Northwest Institute of Plateau Biology & Institute of Sanjiangyuan National Park, Chinese Academy of Sciences, Xining 810001, China; lengzhenning@nwipb.cas.cn (Z.L.); pangzhe@nwipb.cas.cn (Z.P.); hzj@stu.dali.edu.cn (Z.H.); 2 University of Chinese Academy of Sciences, Beijing 100049, China; 3 Qinghai Provincial Key Laboratory of Crop Molecular Breeding, Xining 810001, China

**Keywords:** *Saxifraga* L., complete chloroplast genome, European alps, comparative analysis, phylogenetic analysis

## Abstract

Complete chloroplast genome sequences are widely used in the analyses of phylogenetic relationships among angiosperms. As a species-rich genus, species diversity centers of *Saxifraga* L. include mountainous regions of Eurasia, such as the Alps and the Qinghai–Tibetan Plateau (QTP) *sensu lato*. However, to date, datasets of chloroplast genomes of *Saxifraga* have been concentrated on the QTP species; those from European Alps are largely unavailable, which hinders comprehensively comparative and evolutionary analyses of chloroplast genomes in this genus. Here, complete chloroplast genomes of 19 *Saxifraga* species were *de novo* sequenced, assembled and annotated, and of these 15 species from Alps were reported for the first time. Subsequent comparative analysis and phylogenetic reconstruction were also conducted. Chloroplast genome length of the 19 *Saxifraga* species range from 149,217 bp to 152,282 bp with a typical quadripartite structure. All individual chloroplast genome included in this study contains 113 unique genes, including 79 protein-coding genes, four rRNAs and 30 tRNAs. The IR boundaries keep relatively conserved with minor expansion in *S. consanguinea*. mVISTA analysis and identification of polymorphic loci for molecular markers shows that six intergenic regions (*ndhC*-*trnV*, *psbE*-*petL*, *rpl32*-*trnL*, *rps16*-*trnQ*, *trnF*-*ndhJ*, *trnS*-*trnG*) can be selected as the potential DNA barcodes. A total of 1204 SSRs, 433 tandem repeats and 534 Large sequence repeats were identified in the 19 *Saxifraga* chloroplast genomes. The codon usage analysis revealed that *Saxifraga* chloroplast genome codon prefers to end in A/T. Phylogenetic reconstruction of 33 species (31 *Saxifraga* species included) based on 75 common protein coding genes received high bootstrap support values for nearly all identified nodes, and revealed a tree topology similar to previous studies.

## 1. Introduction

The large arctic–alpine genus *Saxifraga* L. (Saxifragaceae), which comprises 450–500 species, is widely distributed across the Northern Hemisphere [1]. At least two mountainous regions can be recognized as species diversity centers of *Saxifraga*: the Alps in Europe [2] and the Qinghai–Tibetan Plateau *sensu lato* (including Himalayas, Henduan Mts., and plateau platform) in Asia [3]. Considering considerable species richness and habitat diversity, as well as a corresponding high level of morphological, physiological and life cycle diversity [4], *Saxifraga* offers an ideal system to reveal potential driving factors for the formation of high levels of biodiversity in association with mountains, as in [1] or [5,6,7].

A finely resolved and well supported phylogenetic topology of a given taxonomic group is essential to conduct further evolutionary studies, such as biogeographic analysis, trait reconstruction and ploidy evolution [8]. However, phylogenetic relationships of lineages which have experienced recent rapid radiation could not be well resolved by traditionally universal DNA markers [9]. As for the phylogeny of *Saxifraga*, although the infrageneric skeleton frame at sectional level has been revealed to be based on two universal markers [10], phylogenetic relationships within sections were not well resolved [4,10], partly due to rapid radiation [1]. Because of the small size, relatively conservative structure and maternal inheritance, chloroplast genomes have been widely applied to plant phylogeny and evolution, e.g., in [11,12,13]. To date, the large chloroplast genome dataset of *Saxifraga* has been predominantly focused on *S*. sect. *Ciliatae* Haw. [14], the most species-rich section whose center of diversity is the QTP *sensu lato*. The phylogenetic relationship was well resolved within this recent-radiation section, indicating a potentially good performance of chloroplast genome data on the reconstruction of phylogenetic relationships at an infra-sectional level of *Saxifraga*. However, chloroplast genomes from the remaining sections are limited, even absent, especially those from the Alps, which hinders reliable phylogenetic reconstructions both at generic and infra-sectional levels. On the other hand, although the structure and gene content of chloroplast genomes are conserved in most angiosperms, extensive gene losses and large inversions have been detected in several lineages, such as Gentianaceae [15,16], Asteraceae [17,18] and Leguminosae [19,20]. Previous studies [9,14] have confirmed high conservation in structural organization, gene arrangement and gene content in chloroplast genomes of *S*. sect. *Ciliatae*. However, whether this is the case at the generic level of *Saxifraga* is still unclear due to the asymmetry in the available data of chloroplast genomes between the QTP and Alps.

In this study, 19 chloroplast genomes in *Saxifraga* were *de novo* sequenced, assembled and annotated, representing 7 of the 13 sections [10]. Among the 19 species included in this study, 17 are from the European Alps and 15 of which are sequenced for the first time. Comparative analyses were conducted to reveal the molecular evolution of chloroplast genomes in *Saxifraga*. Meanwhile, the performance of phylogenetic reconstruction was tested based on the 19 newly generated sequences combined with additional 12 *Saxifraga* species downloaded from NCBI. This study will enlarge the dataset of *Saxifraga* chloroplast genomes, improve the recognition of chloroplast genome structure and provide a general phylogeny outline of the whole genus.

## 2. Results

### 2.1. Comparative Chloroplast Genomes of Saxifraga Species

#### 2.1.1. General Feature Comparison

**Figure 1:**
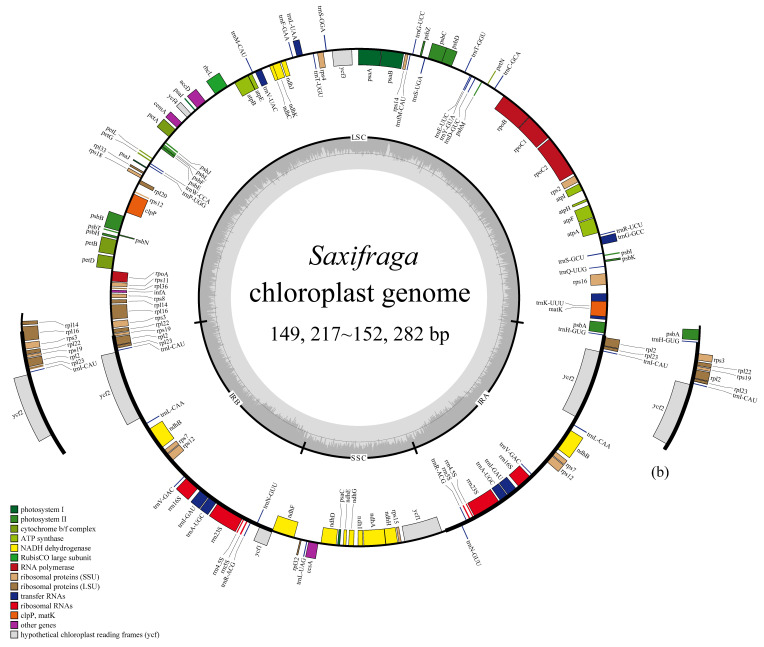
Chloroplast genome structures of 19 *Saxifraga* chloroplast genomes; (**b**) shows the difference in the *S. consanguinea* chloroplast genome compared with the remaining 18 species.

The chloroplast genomes of the 19 *Saxifraga* species have a typical quadripartite structure and contain a large single copy (LSC), a small single copy (SSC), and two copies of inverted repeat (IR) regions (Figure 1). The size of the 19 chloroplast genomes ranges from 149,217 bp (*S*. *aphylla* Sternb.) to 152,282 bp (*S*. *rotundifolia* L.) (Table 1). *Saxifraga consanguinea* W. W. Sm. has the largest IR region (27,220 bp), but the smallest LSC (78,948) and SSC (16,659) among the 19 chloroplast genomes. The largest LSC (83,618) and SSC (17,352) occur in *S. rotundifolia*, while the smallest IR (25,378) occurs in *S. androsacea* L. (Table 1). The total GC content varies from 37.66% to 37.84%, and GC contents of LSC, SSC and IR range from 35.67% to 36.16%, 31.70% to 32.10% and 42.04% to 42.92%, respectively (Table 1). Despite size variations, the GC-contents are similar among the 19 *Saxifraga* species in the whole genome, LSC, SSC and IR regions.

Each of the 19 newly generated *Saxifraga* chloroplast genomes contains 113 unique genes, among which 79 genes are PCGs, 4 are rRNA genes and 30 are tRNA genes (Table 2). Among the 113 unique genes, 7 PCGs (*ndhB*, *rpl2*, *rpl23*, *rps12*, *rps7*, *ycf2*, *ycf1*), all the 4 rRNA genes and 7 tRNA genes (*trnA*-*UGC*, *trnI*-*CAU*, *trnI*-*GAU*, *trnL*-*CAA*, *trnN*-*GUU*, *trnR*-*ACG*) are completely or partially duplicated in the IR region, which results in a total number of 131 genes in all of the remaining 18 genomes except *S. consanguinea* (Table 2). As for the chloroplast genome of *S. consanguinea*, 3 additional PCGs (*rps19*, *rpl22*, *rps3*) are also duplicated in the IR region, leading to a total number of 134 genes in this species. By counting the introns in genes, 2 genes have two introns and 15 genes have one intron (Table 2). The *rps12* gene is trans-spliced, with its 5′-end exon located in the LSC region and 3′-end exon duplicated in the IRs. Regarding the function of genes, 45 of them take part in photosynthesis, 59 in self-replication and 9 in other functions (Table 2).

#### 2.1.2. IR Boundary Variation Analysis

**Figure 2:**
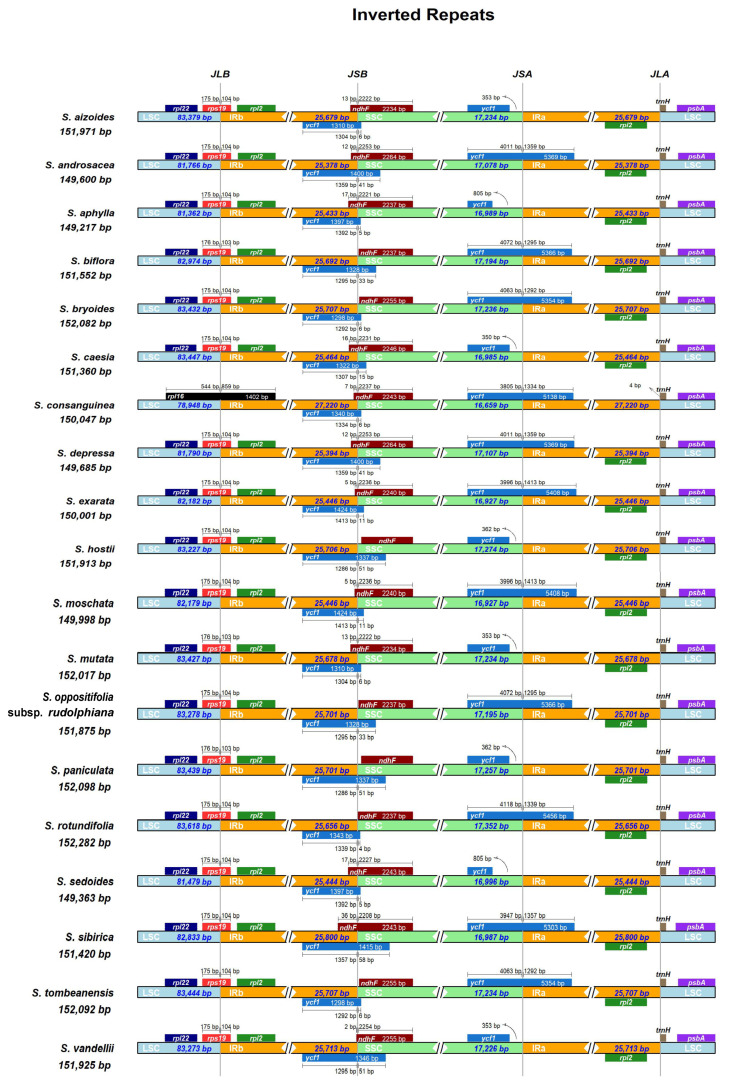
Comparison of boundaries of LSC, IR and SSC regions among the 19 *Saxifraga* chloroplast genomes. Selected genes are indicated by boxes along the genome. Genes above the genome lines indicate their transcriptions in the forward direction, while below is the reverse direction. *JLB* junction between LSC and IRB, *JSB* junction between SSC and IRB, *JSA* junction between SSC and IRA, *JLA* junction between LSC and IR.

The boundaries between LSC, IR and SSC, as well as the adjacent genes, were compared across the 19 *Saxifraga* chloroplast genomes (Figure 2). The most massive expansion of IR boundaries was detected between LSC and IRB regions in *S. consanguinea*, which expended to the *rpl16* gene in LSC. This resulted in a duplication of three additional PCGs (*rps19*, *rpl22*, *rps3*) compared to the remaining chloroplast genomes, leading to the largest IR region (27,220 bp), but the smallest LSC (78,948) and SSC (16,659), in *S. consanguinea* (Table 1, Figure 2). However, for the remaining 18 chloroplast genomes, the LSC/IRB junctions were all located within the coding region of *rps19*. The expansion/contraction of IRs were also detected in junctions of IRB/SSC which fell into the *ycf1* pseudogene and/or *ndhF* gene, as well as SSC/IRA which was located within the *ycf1* gene but with different extensions (Figure 2). The IRA/LSC border of the 19 chloroplast genomes was mainly conserved, exactly matching or being a few basepairs ahead of the start codon of *trnH* (Figure 2).

#### 2.1.3. Polymorphic Analysis Among Chloroplast Genome Sequences

**Figure 3:**
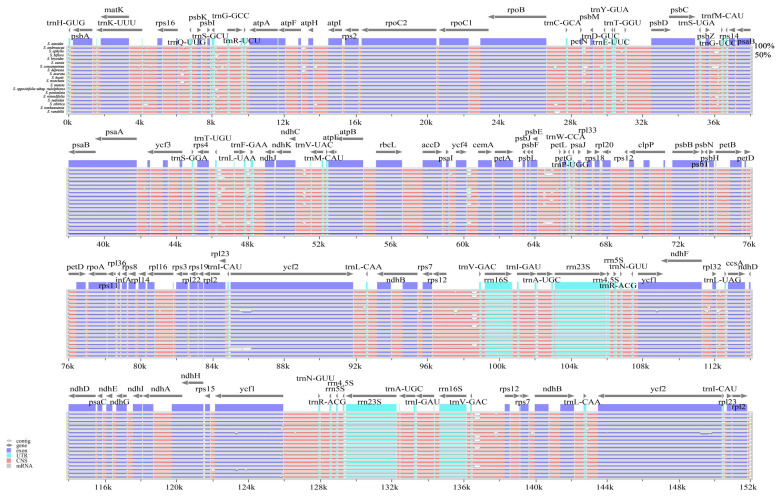
Global chloroplast genome alignments for the 19 *Saxifraga* species using the mVISTA program, with *S. aizoides* as the reference. The *Y*-axis shows the range of sequence identity (50–100%).

**Figure 4:**
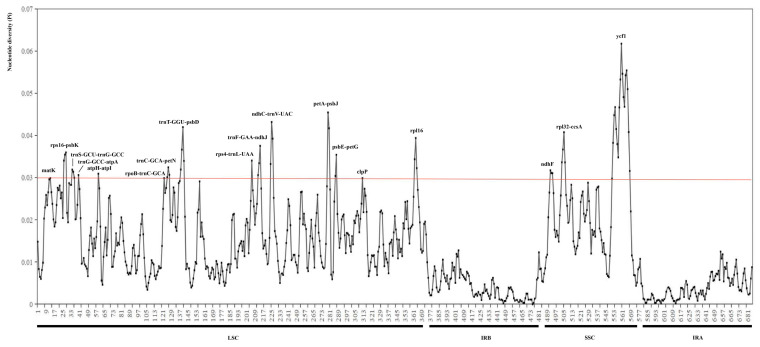
Nucleotide variability values compared between the 19 *Saxifraga* chloroplast genomes using the window sliding analysis. *X*-axis indicates the position of the midpoint of the window, while *Y*-axis indicates the nucleotide diversity of each window.

The overall sequence identity of the 19 chloroplast genomes of *Saxifraga* was plotted using mVISTA with the annotation of the *S. aizoides* L. chloroplast genome as the reference (Figure 3). The results showed that the *Saxifraga* chloroplast genomes exhibited a high level of sequence synteny, suggesting a conserved evolutionary pattern. Nucleotide variability values were calculated using the window sliding analysis, as implemented in DnaSP (Figure 4). A total of 685 highly variable regions were identified among the 19 chloroplast genomes, of which 18 regions exhibited a nucleotide diversity value (π) higher than 0.03. As a whole, the IR regions are less divergent compared to the LSC and SSC region, and the coding regions are more conserved than the intergenic spacers and introns. Among the 18 most variable regions, 13 locations (*rps16*-*psbK*, *trnS*-*GCU*-*trnG*-*GCC*, *trnG*-*GCC*-*atpA*, *atpH*-*atpI*, *rpoB*-*trnC*-*GCA*, *trnC*-*GCA*-*petN*, *trnT*-*GGU*-*psbD*, *rps4*-*trnL*-*UAA*, *trnF*-*GAA*-*ndhJ*, *ndhC*-*trnV*-*UAC*, *petA*-*psbJ*, *psbE*-*petG*, *rpl32*-*ccsA*) are intergenic regions, and 5 locations (*matK*, *clpP*, *rpl16*, *ndhF*, *ycf1*) are protein coding regions. The *ycf1* pseudogene, which is located at the boundary of SSC/IRA, had the highest nucleotide variation (π = 0.06174). Its high polymorphism may relate to the expansion/contraction of IR regions. These highly variable regions can be used as candidates of the DNA barcodes for *Saxifraga*.

#### 2.1.4. Repeat Sequences Analysis

**Figure 5:**
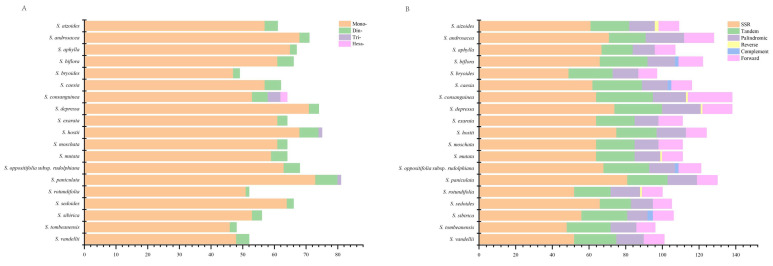
Analyses of repeat sequences in the 19 *Saxifraga* chloroplast genomes. (**A**) Number of SSRs types. (**B**) Number of SSRs, tandem repeats and LSRs.

A total of 1204 SSRs were identified among the 19 *Saxifraga* chloroplast genomes. The mono-, di-, tri- and hexa-nucleotide repeats accounted for 93.11%, 5.82%, 0.77% and 0.31%, respectively. No tetra- or penta-nucleotide repeats were detected in the 19 *Saxifraga* chloroplast genomes included in this study (Figure 5). The two dominant SSRs motif types were A/T and AT/TA. *S. paniculata* Mill. possessed the highest number of SSRs, while *S. consanguinea* had the highest abundance of SSRs types (Figure 5). A total of 433 Tandem repeats and 534 large sequence repeats (LSRs; ≥30 bp and Hamming distance = 3) were identified among the 19 *Saxifraga* chloroplast genomes. Both of these were most abundant in the *S. consanguinea* chloroplast genome compared to the remaining genomes (Figure 5).

#### 2.1.5. Codon Usage Analysis

**Figure 6:**
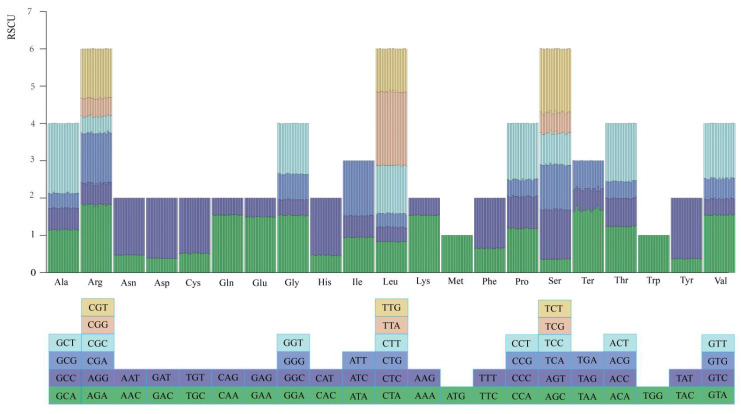
Results of relative synonymous codon usage (RSCU) analysis and codon frequency of the 20 amino acids encoded in all PCGs of the 19 *Saxifraga* chloroplast genomes. The histogram above each amino acid shows codon usage. Colors in the column graph reflected codons in the same colors shown below the figure. Ala: alanine; Arg: arginine; Asn: asparagine; Asp: aspartate; Cys: cysteine; Gln: glutamine; Glu: glutamic; Gly: glycine; His: histidine; Ile: isoleucine; Leu: leucine; Lys: lysine; Met: methionine; Phe: phenylalanine; Pro: proline; Ser: serine; Ter: terminal codon; Thr: threonine; Trp: tryptophan; Tyr: tyrosine; Val: valine. From left to right: 19 *Saxifraga* species.

Condon usage analysis revealed that the 19 *Saxifraga* species contained 61 codons encoding 20 amino acids, as well as three stop codons (Figure 6). The total number of codons of the 19 species ranged from 19,897 in *S. sedoides* L. to 20,709 in *S. rotundifolia* L. Leucine is the most frequently found amino acid (40,377), and cysteine is the rarest (4382). The RSCU value calculation result showed 30 codons with an RSCU value greater than 1, suggesting a higher codon usage frequency than expected, while there were 32 codons with an RSCU value less than 1, indicating a lower codon usage frequency. Among the codons with RSCU value > 1, 12 codons ended with A, 16 codons ended with T, and only 1 codon ended with G, indicating an A/T ending preference of *Saxifraga* chloroplast genome codons.

### 2.2. Phylogenetic Analysis

**Figure 7:**
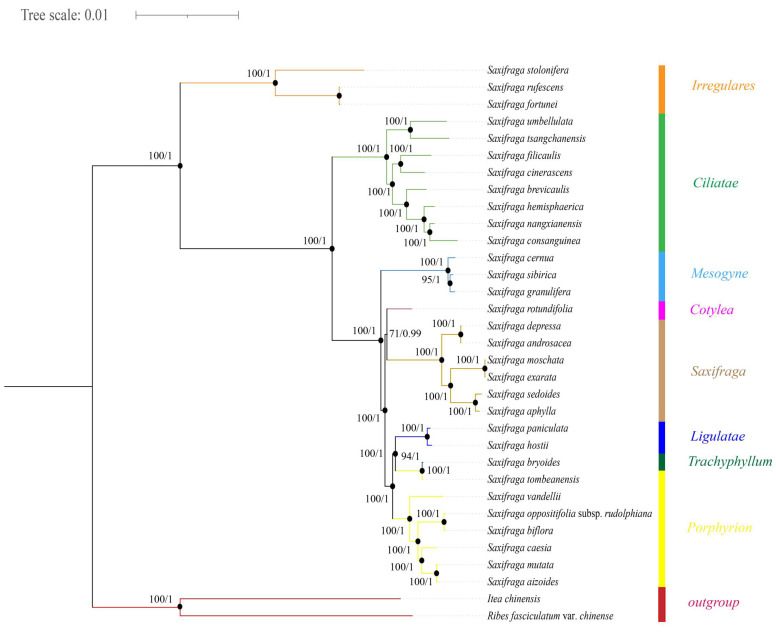
The phylogenetic tree of *Saxifraga* constructed by means of maximum likelihood and Bayesian interference methods. Numbers at the nodes represent ML bootstrap (**left**) and BI posterior probability (**right**) values. Different colors represent different groups.

In total, 75 common genes (*accD*, *atpA*, *atpB*, *atpE*, *atpF*, *atpH*, *atpI*, *ccsA*, *cemA*, *infA*, *matK*, *ndhA*, *ndhB*, *ndhC*, *ndhD*, *ndhE*, *ndhF*, *ndhG*, *ndhH*, *ndhI*, *ndhJ*, *ndhK*, *petA*, *petB*, *petD*, *petG*, *petL*, *petN*, *psaA*, *psaB*, *psaC*, *psaI*, *psaJ*, *psbA*, *psbB*, *psbC*, *psbD*, *psbE*, *psbF*, *psbH*, *psbI*, *psbJ*, *psbK*, *psbL*, *psbM*, *psbT*, *psbZ*, *rbcL*, *rpl2*, *rpl14*, *rpl16*, *rpl20*, *rpl22*, *rpl23*, *rpl32*, *rpl33*, *rpl36*, *rpoA*, *rpoB*, *rpoC1*, *rpoC2*, *rps2*, *rps3*, *rps4*, *rps7*, *rps8*, *rps11*, *rps12*, *rps14*, *rps15*, *rps16*, *rps18*, *rps19*, *ycf1*, *ycf2*) were extracted, and phylogenetic relationships were reconstructed using *R. fasciculatum* var. *chinense* and *I. chinensis* as outgroups. Referring to the description of phylogenetic results by Tkach [10], firstly, three species—*S. stolonifera*, *S. rufescens* and *S. fortunei*—were clustered together, belonging to sect. *Irregulares*. Sect. *Ciliatae* consisted of eight species, including *S. umbellulata*, *S. tsangchanensis*, *S. filicaulis*, *S. cinerascens*, *S. brevicaulis*, *S. hemisphaerica*, *S. nangxianensis* and *S. consanguinea*. Sect. *Mesogyne* included *S. cernua*, *S. sibirica* and *S. granulifera*, while Sect. *Cotylea* was represented solely by *S. rotundifolia*. Six species, including *S. depressa*, *S. androsacea*, *S. moschata*, *S. exarata*, *S. sedoides* and *S. aphylla*, were assigned to sect. *Saxifraga*. Sect. *Ligulatae* contained *S. paniculata* and *S. hostii*. Sect. *Trachyphyllum* was represented by *S. bryoides*. Sect. *Porphyrion* encompassed seven species, including *S. tombeanensis*, *S. vandellii*, *S. oppositifolia* subsp. *rudolphiana*, *S. biflora*, *S. caesia*, *S. mutata* and *S.aizoides*. The ML and BI tree topologies were highly congruent and were consistent with previous studies [10]. The BS and PP values were fairly high, all but three nodes presented a BS value of 100% and all nodes of PP values reached 1, with the exception of one node (Figure 7).

## 3. Discussion

### 3.1. Chloroplast Genome Structure Variation Within the 19 Saxifraga Species

In general, the gene content and gene organization of angiosperm chloroplast genomes are highly conserved compared to nuclear and mitochondrial genomes [21]. Because of the high stability and conservatism, the whole chloroplast genome has been widely used in plant species identification, population genetics, genome evolution and phylogenetic studies [22]. However, the phenomena of gene rearrangement [17], large fragment loss [16] and even structural variations [23] still exist in some lineages. In this study, the complete chloroplast genomes of 19 *Saxifraga* species were *de novo* sequenced and analyzed. Among them, 15 species from the European Alps have been sequenced for the first time. The genome structure of the 19 *Saxifraga* species is consistent with those of most terrestrial plants, and the size (ranging from 149,217 bp to 152,282 bp) falls well into the range of 120–160 kb of angiosperm chloroplast genomes [24]. The GC contents of the 19 chloroplast genome sequences (37.66–37.84%) were similar to the average of sequenced land plants (37.6%) [25], and the IR regions contain the highest GC content, which is in line with most angiosperm plants. Structure analyses of these newly generated chloroplast genomes show high conservation in structural organization, gene arrangement and gene content. Large structural variation [23], large fragments inversion/loss [16,17] and gene rearrangement [26] are not detected in chloroplast genomes generated in this study, which is congruent with previous studies [9,14]. To date, the largest chloroplast genome dataset of *Saxifraga* was focused on sect. *Ciliatae*, whose diversity center is the QTP *sensu lato* [14]. Nearly one hundred chloroplast genomes are available for this largest section of *Saxifraga*, and genome structure has been confirmed to be rather conservative [14]. In this study, chloroplast genomes of 15 *Saxifraga* species from the Alps were generated for the first time, which can give us a relatively comprehensive scope of chloroplast genome evolution at the generic level of *Saxifraga*. Although chloroplast genome data of European species are still urgently needed, our results, combined with those published data, may suggest a relatively conservative evolutionary history of chloroplast genomes at the generic level of *Saxifraga*.

The expansion and contraction of the chloroplast genome is a common evolutionary phenomenon in plants [27]. In angiosperms, the expansion/contraction of the IR boundaries of chloroplast genomes often result in different levels of genome size variation, gene duplication or production of pseudogenes [28,29,30]. Because of the expansion of the IR boundary, *S*. *consanguinea* has the biggest length in IR regions among 19 *Saxifraga* species. The large IRs of the plastomes are hypothesized to contribute to plastome stabilization because their absence often coincides with severe changes in gene order [31]. However, the SSC and LSC regions are the smallest of *S. consanguinea*, and kept the total length relatively consistent. The balance between the SC regions and IR regions might be the reason for the stability in length. Although an extension of IR regions was detected in *S. consanguinea*, it seems that IR boundaries are much conservative among *Saxifraga* species: 18 out of the 19 chloroplast genomes share the same type of IR boundaries. This was also revealed by Yuan et al. [14], in which 88 of the 94 sect. *Ciliatae* chloroplast genomes shared the same type of IR boundaries, and only 6 species showed IR expansion/contraction. Pseudogenes play an essential role in gene expression regulation and genome evolution [32]. Two pseudogenes of *rps19* and *ycf1* were found in this study, coincident with the results revealed in *Aconitum* [33]. Meanwhile, pseudogene *rps19* was found in 18 of the 19 *Saxifraga* species, while *ycf1* was detected in 8 species, indicating a species specificity of pseudogenes among species. Meanwhile, *ycf1* has been confirmed to be associated with high altitude [34], and so the duplication in IR regions might contribute to its adaptation.

Although chloroplast genomes in this study exhibit high conservation in genome structure and IR boundary, regions with high sequence polymorphisms (e.g., *ndhC*-*trnV*, *psbE*-*petL*, *rpl32*-*trnL*, *rps16*-*trnQ*, *trnF*-*ndhJ*, *trnS*-*trnG*, *ycf1*) are observed among the 19 chloroplast genomes. These highly divergent regions are also revealed at the section level of sect. *Ciliatae* [14], as well as at the family level of Saxifragaceae [9], and can be used as candidate barcoding regions for species identification, population genetics and phylogenetics of *Saxifraga*.

Due to high levels of variations, chloroplast SSRs (≥10 bp) play an important role in polymorphism investigations, population genetics and phylogenetic analyses [35,36,37,38,39]. In this study, the number of SSRs ranges from 48 to 81 among the 19 *Saxifraga* species, which shows a moderate level compared with other species of angiosperms [17,40,41]. Most of these SSRs are located in the LSC region, followed by the SSC and IR regions. The high level of A/T content and the predominance of mononucleotide repeats are the significant features in chloroplast SSRs of the 19 *Saxifraga* species, which may reflect a common phenomenon in angiosperms [15,42]. In addition, the high AT content of chloroplast SSRs may be caused by the main contribution of poly (A), poly (T) or poly (AT) repeats in the non-coding regions of the single-copy regions, especially in the LSC region [43]. Furthermore, SSRs detected in this study are mainly distributed in the non-coding region, including intergenetic regions and gene introns. In summary, the range of SSR numbers, frequency of different SSRs types and the distribution patterns of SSRs across the 19 chloroplast genomes are comparable with those in sect. *Ciliatae* [9,14]. Long repeats play an essential role in the whole-chloroplast genome variation, expansion and rearrangement [44]. We identified 17–31 tandem repeats, as well as 22–43 large sequence repeats, among the 19 chloroplast genomes, indicating a relative abundance of long repeats in *Saxifraga* chloroplast genomes. These SSRs and long repeats usually exhibit high levels of variation, and thus provide potential candidates for the development of molecular markers for future evolutionary and genetic diversity studies in *Saxifraga*.

Codon usage preference has been documented as one of the evolutionary features in many organisms [45]. Our results revealed that the number of codon types and the frequency of amino acids, as well as codon usage preference, are similar to those revealed in sect. *Ciliatae* [14].

Despite the limited number of species included in this study, it represents 7 of the 13 sections of *Saxifraga* [10]. According to our results in this study, together with previous studies [9,14,46], chloroplast genomes of *Saxifraga* may have experienced a conservative evolutionary history, as proven by (i) high conservation in structural organization, gene arrangement and gene content; (ii) many conservative IR boundaries; (iii) similarity in SSRs numbers, types frequencies and distribution patterns; and (iv) comparable codon types and codon usage preference. However, more chloroplast genomes of *Saxifraga* species, especially those from the European Alps, are needed to test a comprehensive chloroplast genome evolution in this genus.

### 3.2. Phylogeny of Saxifraga Species

To date, the most comprehensive investigation on the phylogenetic relationships at the generic level of *Saxifraga* was that conducted by Tkach et al. [10], employing a balanced sampling strategy of 254 *Saxifraga* species and two universal DNA markers (nrDNA ITS and plastid *trnL*-*F*). A backbone framework of at least 13 sections and 9 subsections was recognized within *Saxifraga* [10]. However, due to the recent rapid radiation of this species-rich genus [1], some difficulties may occur during phylogenetic reconstruction when using only a few universal DNA markers. On the one hand, rapid radiations are usually associated with low genetic differentiation between closely related clades or species, making phylogenetic reconstruction difficult. On the other hand, limited informative sites when using only a few DNA markers might not reveal a well-resolved phylogenetic relationship. This is the case in the phylogenetic study of *Saxifraga* at the moment. Firstly, some of the major clades within *Saxifraga* as recognized by Tkach et al. [10] are not well supported, leading to an unclear placement or relationship on the phylogenetic tree. Secondly, the phylogenetic relationships at the infra-section/subsection level are not well resolved, especially for those with high species richness, such as sect. *Saxifraga* and sect. *Porphyrion* Tausch. Thirdly, taxonomic positions of some *Saxifraga* species, such as *S. odontophylla* Wall. ex Sternb. and *S. nana* Engl., are still ambiguous. Complete chloroplast genome sequences seem to offer an opportunity to resolve problems in *Saxifraga* phylogenetic study, as mentioned above. Yuan et al. [14] employed ca. 100 chloroplast genomes to investigate the infra-section relationships of sect. *Ciliatae*, the most species-rich section which has experienced recent radiation. Phylogenetic relationships within sect. *Ciliatae* using complete chloroplast genome sequences are better resolved and have higher support values compared to that using few DNA markers [4]. In this study, 31 *Saxifraga* species from eight sections are employed to test the performance of the complete chloroplast genome on the resolution of phylogenetic relationships of *Saxifraga*. The resolution and support values as revealed by phylogenetic tree are extremely high even at the tip nodes, suggesting a good performance of complete chloroplast genome sequences on the resolution of phylogenetic relationships of *Saxifraga* at infra-section/subsection level.

In conclusion, our study adds new complete chloroplast genome sequences to the molecular dataset of *Saxifraga*. The evolutionary history of chloroplast genomes of *Saxifraga* may be conservative. Complete chloroplast genome sequences seem to offer an opportunity to resolve phylogenetic relationships at the infra-section/subsection level of *Saxifraga*, but more new sequences should be generated.

## 4. Materials and Methods

### 4.1. Sample Collection, DNA Extraction, and Sequencing

A total of 19 species were sampled, of which 17 were from the Alps and 2 from Sino-Himalaya (Table 3). The 19 species represent 7 of the 13 sections proposed by Gornall [47] and Tkach et al. [10]. Leaves were collected from a single individual, then dried in silica gel. The voucher specimen was deposited in the herbarium of the University of Leicester, Leicester, England, and the Northwest Institute of Plateau Biology (HNWP), Xining, Qinghai, China. Total genomic DNA was extracted from silica-dried leaves by the DNA quick extraction system (DP321) according to the manufacturer’s protocol (Tiangen Biochemical Technology Co., Ltd., Beijing, China). Total DNA was then randomly fragmented with the Covaris ultrasonic crusher. A series of steps was performed to complete library construction, such as end repair and phosphorylation, a-tail addition, sequencing connector addition, purification and PCR amplification. Finally, the qualified libraries were pooled into flowcells. Paired-end sequencing from both ends of 150 bp fragments was performed on the Illumina NovaSeq 6000 platform at the BENAGEN company in Wuhan, China, to generate ~5 Gb data for each individual. We used fastp v.0.23.1 [48] to filter raw sequence reads when (i) the N content in any read was more than 10% of the base; (ii) the number of low-quality (Q ≤ 5) bases in any read exceeded 50%; and (iii) any read contained the adapter content [49].

### 4.2. Chloroplast Genome Assembly and Annotation

High-quality clean reads were assembled using GetOrganelle v.1.7.5 [50] with the default parameters and *S. sinomontana* J-T. Pan & Gornall as a reference genome (GenBank accession no. MN104589) [9]. Circular genomes were annotated using CPGAVAS2 (http://47.96.249.172:16019/analyzer/home (accessed on 22 January 2025)) [51]. The preliminarily annotation results may have some problems, such as genes with nonstandard start or stop codons or genes with an internal stop codon. The correctness was checked using CPGView [52]. Finally, well-annotated sequences were then submitted to OGDRAW (https://chlorobox.mpimp-golm.mpg.de/OGDraw.html (accessed on 20 December 2024)) for chloroplast genome visualization [53]. All the complete chloroplast genome sequences were deposited into GenBank, with the accession numbers PV423509 and PV426729-426746.

### 4.3. Comparative Analysis of Chloroplast Genomes

The length, GC content of the total sequence, LSC region, SSC region and IR regions, as well as numbers of protein-coding genes, tRNA genes and rRNA genes, were calculated by command lines using Qt Console v5.5.1. The gene types were counted by CPStools [54]. A comparison of junction sites of LSC, IR and SSC regions was implemented using the program IRscope (http://msgvd.genehub.com.cn/IRscope/ (accessed on 27 November 2024)) [55]. The percentage of sequence identity was analyzed and plotted using the program mVISTA (https://genome.lbl.gov/vista/mvista/submit.shtml (accessed on 18 December 2024)) [56], with an alignment algorithm of LAGAN. To identify high variable regions, the 19 chloroplast genomes were aligned using MAFFT v.7 [57] in PhyloSuite [58] with default parameters. The number of polymorphic sites and nucleotide variability (Pi) were evaluated using a sliding window with 200 bp step size and a 600 bp window length implemented in DnaSP v5.10.1 [59]. Simple sequence repeats (SSRs) were detected using MISA (https://webblast.ipk-gatersleben.de/misa/ (accessed on 8 January 2025)) [60]. Tandem repeats were identified with Tandem Repeats Finder v4.09 [61]. Large sequence repeats (LSRs) were identified using REPuter with hamming distance = 3, maximum computed repeats of 50 bp and minimum repeat size of 30 bp [62]. The codon usage and the relative synonymous codon usage (RSCU) value were estimated using CPStools [54]. To reduce sampling error, protein-coding genes (PCGs) shorter than 300 bp and the genes utilizing non-standard start codons were filtered. The RSCU plot was created with the online tool Genepioneer (http://112.86.217.82:9929 (accessed on 6 January 2025)). An RSCU value greater than 1 indicates a higher frequency of codon usage, while a value less than 1 indicates a lower frequency [63].

### 4.4. Phylogenetic Analysis

The performance of phylogenetic reconstruction was tested based on the 19 newly generated sequences combined with an additional 12 *Saxifraga* species downloaded from NCBI, using *Ribes fasciculatum* Siebold & Zucc. var. *chinense* Maxim. (MH191388) and *Itea chinensis* Hook. f. & Arn. (NC_037884) as the outgroups. The 12 downloaded *Saxifraga* species include (i) *S. cinerascens* Engl. & Irmsch. (NC_070452), *S. nangxianensis* J. T. Pan (NC_070492), *S. filicaulis* Wall. ex Ser. (NC_070461), *S. hemisphaerica* Hook. f. & Thoms. (NC_070471), *S. tsangchanensis* Franch. (NC_070517), *S. umbellulata* Hook. f. & Thoms. (NC_070518) and *S. brevicaulis* Harry Sm. (NC_070447) from sect. *Ciliatae*; (ii) *S. fortunei* Hook. f. (NC_070463), *S. rufescens* Balf. f. (NC_070504) and *S. stolonifera* Curt. (NC_037882) from sect. *Irregulares* Haw.; and (iii) *S. cernua* L. (NC_070450) and *S. granulifera* Harry Sm. (NC_070468) from sect. *Mesogyne* Sternb. was downloaded from the NCBI database. In total, 31 *Saxifraga* species from eight sections, plus two outgroups, were included to conduct phylogenetic analysis. Common protein coding genes (PCGs) were extracted using PhyloSuite and concatenated into a single matrix for each species. The concatenated sequences were then aligned using MAFFT. Phylogenetic relationships were reconstructed by means of maximum likelihood (ML) and Bayesian inference (BI) using IQ-TREE [64] and MrBayes [65], respectively, as implemented in PhyloSuite. The best-fitting models were GTR + F+I + G4 according to BIC and GTR + I + G, separately. Bootstrap support (BS) values and posterior probabilities (PP) were calculated using 1000 replications. Phylogenetic trees were visualized and adjusted with iTOL v.6 [66].

## Figures and Tables

**Table 1:** General characteristics of the 19 *Saxifraga* chloroplast genomes.

Species	Length (bp)				GC Content (%)				Gene (Unique Gene) Number			
	Total	LSC	SSC	IR	Total	LSC	SSC	IR	Total	CDS	tRNA	rRNA
*S. aizoides*	151,971	83,379	17,234	25,679	37.75	35.81	31.99	42.82	131 (113)	86 (79)	37 (30)	8 (4)
*S. androsacea*	149,600	81,766	17,078	25,378	37.69	35.72	31.71	42.88	131 (113)	86 (79)	37 (30)	8 (4)
*S. aphylla*	149,217	81,362	16,989	25,433	37.77	35.81	31.90	42.86	131 (113)	86 (79)	37 (30)	8 (4)
*S. biflora*	151,552	82,974	17,194	25,692	37.76	35.88	31.84	42.78	131 (113)	86 (79)	37 (30)	8 (4)
*S. bryoides*	152,082	83,432	17,236	25,707	37.76	35.77	32.09	42.90	131 (113)	86 (79)	37 (30)	8 (4)
*S. caesia*	151,360	83,447	16,985	25,464	37.74	35.79	32.08	42.82	131 (113)	86 (79)	37 (30)	8 (4)
*S. consanguinea*	150,047	78,948	16,659	27,220	37.84	36.16	32.10	42.04	134 (113)	89 (79)	37 (30)	8 (4)
*S. depressa*	149,685	81,790	17,107	25,394	37.67	35.70	31.70	42.86	131 (113)	86 (79)	37 (30)	8 (4)
*S. exarata*	150,001	82,182	16,927	25,446	37.77	35.78	32.00	42.92	131 (113)	86 (79)	37 (30)	8 (4)
*S. hostii*	151,913	83,227	17,274	25,706	37.78	35.85	31.94	42.87	131 (113)	86 (79)	37 (30)	8 (4)
*S. moschata*	149,998	82,179	16,927	25,446	37.77	35.78	31.99	42.92	131 (113)	86 (79)	37 (30)	8 (4)
*S. mutata*	152,017	83,427	17,234	25,678	37.74	35.80	31.98	42.82	131 (113)	86 (79)	37 (30)	8 (4)
*S. oppositifolia* subsp. *rudolphiana*	151,875	83,278	17,195	25,701	37.71	35.81	31.82	42.77	131 (113)	86 (79)	37 (30)	8 (4)
*S. paniculata*	152,098	83,439	17,257	25,701	37.77	35.83	31.95	42.86	131 (113)	86 (79)	37 (30)	8 (4)
*S. rotundifolia*	152,282	83,618	17,352	25,656	37.80	35.90	32.05	42.85	131 (113)	86 (79)	37 (30)	8 (4)
*S. sedoides*	149,363	81,479	16,996	25,444	37.76	35.81	31.85	42.85	131 (113)	86 (79)	37 (30)	8 (4)
*S. sibirica*	151,420	82,833	16,987	25,800	37.66	35.67	31.81	42.77	131 (112)	86 (79)	37 (30)	8 (4)
*S. tombeanensis*	152,092	83,444	17,234	25,707	37.76	35.76	32.09	42.91	131 (113)	86 (79)	37 (30)	8 (4)
*S. vandellii*	151,925	83,273	17,226	25,713	37.76	35.79	32.08	42.86	131 (113)	86 (79)	37 (30)	8 (4)

**Table 2:** Genes present in the 19 *Saxifraga* chloroplast genomes.

Category	Gene Group	Gene Name
Photosynthesis	Subunits of photosystem I	*psaA*, *B*, *C*, *I*, *J*, *ycf3* ^a^
	Subunits of photosystem II	*psbA*, *B*, *C*, *D*, *E*, *F*, *H*, *I*, *J*, *K*, *L*, *M*, *N*, *T*, *Z*
	Subunits of NADH dehydrogenase	*ndhA* ^b^, *B* ^b,c^, *C*, *D*, *E*, *F*, *G*, *H*, *I*, *J*, *K*
	Subunits of cytochrome b/f complex	*petA*, *B* ^b^, *D* ^b^, *G*, *L*, *N*
	Subunits of ATP synthase	*atpA*, *B*, *E*, *F* ^b^, *H*, *I*
	Large subunit of rubisco	*rbcL*
Self-replication	Proteins of large ribosomal subunit	*rpl2* ^c^, *14*, *16* ^b^, *20*, *22* ^e^, *23* ^c^, *32*, *33*, *36*
	Proteins of small ribosomal subunit	*rps2*, *3* ^e^, *4*, *7* ^c^, *8*, *11*, *12* ^b,c,d^, *14*, *15*, *16* ^b^, *18*, *19* ^e^
	Subunits of RNA polymerase	*rpoA*, *B*, *C1* ^b^, *C2*
	Ribosomal RNAs	*rrn4.5* ^c^, *5* ^c^, *16* ^c^, *23* ^c^
	Transfer RNAs	*trnA(UGC)* ^b,c^, *C(GCA)*, *D(GUC)*, *E(UUC)*, *F(GAA)*, *fM(CAU)*, *G(GCC)* ^b^, *G(UCC)*, *H(GUG)*, *I(CAU)* ^c^, *I(GAU)* ^b,c^, *K(UUU)* ^b^, *L(CAA)* ^c^, *L(UAA)* ^b^, *L(UAG)*, *M(CAU)*, *N(GUU)* ^c^, *P(UGG)*, *Q(UUG)*, *R(ACG)* ^c^, *R(UCU)*, *S(GCU)*, *S(GGA)*, *S(UGA)*, *T(GGU)*, *T(UGU)*, *V(GAC)* ^c^, *V(UAC)* ^b^, *W(CCA)*, *Y(GUA)*
Other genes	Maturase	*matK*
	Protease	*clpP* ^a^
	Envelope membrane protein	*cemA*
	Acetyl-CoA carboxylase	*accD*
	c-type cytochrome synthesis gene	*ccsA*
	Translation initiation factor	*infA*
	Proteins of unknown function	*ycf1* ^c^, *ycf2* ^c^, *ycf4*

^a^ Gene containing two introns; ^b^ gene containing a single intron; ^c^ two gene copies in IRs; ^d^ gene divided into two independent transcription units; ^e^ extra two gene copies in IRs of *S. consanguinea.*

**Table 3:** Sampling information of the 19 *Saxifraga* species, as well as the GenBank accession numbers of the generated chloroplast genome sequences.

Taxa	Locality	Latitude (N)	Longitude (E)	Altitude (m)	GenBank Accession No.
*Saxifraga* sect. *Ciliatae* Haw.					
*S. consanguinea* W. W. Sm.	Dari, Qinghai, China	33.44068°	98.66934°	4556	PV426734
Sect. *Cotylea* Tausch					
*S. rotundifolia* L.	Val di Loriva, Italy	45.82570°	10.61749°	1900	PV426742
Sect. *Ligulatae* Haw.					
*S. hostii* Tausch	Passo di Maniva, Italy	45.81642°	10.41266°	1670	PV426737
*S. paniculata* Mill.	Colfosco Calfosch, Italy	46.55071°	11.83100°	1853	PV426741
Sect. *Mesogyne* Sternb.					
*S. sibirica* L.	Shennongjia, Hubei, China	31.44257°	110.27239°	2875	PV426744
Sect. *Porphyrion* Tausch					
*S. aizoides* L.	Grossglockner, Austria	47.07452°	12.74526°	2084	PV423509
*S. biflora* All.	Grossglockner, Austria	47.07452°	12.74526°	2084	PV426731
*S. caesia* L.	Manira Pass, Italy	45.80359°	10.41020°	1636	PV426733
*S. mutata* L.	Magasa, Italy	45.78538°	10.62095°	940	PV426739
*S. oppositifolia* L. subsp. *rudolphiana* (Hornsch. ex W. D. J. Koch) Engl. & Irmsch.	Grossglockner, Austria	47.07452°	12.74526°	2084	PV426740
*S. tombeanensis* Boiss. ex Engl.	Arabba, Italy	46.47297°	11.87086°	2521	PV426745
*S. vandellii* Sternb.	Presolana, Italy	45.96681°	10.04827°	2040	PV426746
Sect. *Saxifraga*					
*S. androsacea* L.	Col Rodela, Italy	46.49837°	11.74894°	2300	PV426729
*S. aphylla* Sternb.	Innsbruck, Austria	47.31284°	11.38437°	2300	PV426730
*S. depressa* Sternb.	Arabba, Italy	46.47297°	11.87086°	2521	PV426735
*S. exarata* Vill.	Lago Bianco, Switzerland	46.40780°	10.02137°	2276	PV426736
*S. moschata* Wulfen	Rudolfshectte, Austria	47.13442°	12.62523°	2313	PV426738
*S. sedoides* L.	Sassolungo, Italy	46.51657°	11.73929°	2685	PV426743
Sect. Trachyphyllum (Gaudin) W. D. J. Koch					
*S. bryoides* L.	Grossglockner, Austria	47.12626°	12.81354°	2121	PV426732

## Data Availability

The original contributions presented in the study are included in the article material; further inquiries can be directed to the corresponding authors.
